# Site-Specifically Modified Circular Ribonucleic Acid Serves as Multitarget miRNA Sponge with Low Immunogenicity

**DOI:** 10.1021/jacs.6c04991

**Published:** 2026-04-11

**Authors:** Yufan Pan, Xin Li, Bini Zhou, Yuan Zhuang, Liangzhi Luo, Chenyou Zhu, Rui Xu, Yifan Jiang, Yuanchen Dong, Ziyang Hao, Dongsheng Liu, Xi Zhang

**Affiliations:** † Department of Chemistry, 12442 Tsinghua University, Beijing 100084, China; ‡ School of Pharmaceutical Sciences, 12517 Capital Medical University, Beijing 100069, China; § CAS Key Laboratory of Colloid Interface and Chemical Thermodynamics, Beijing National Laboratory for Molecular Sciences, Institute of Chemistry, Chinese Academy of Sciences, Beijing 100190, China; ∥ University of Chinese Academy of Sciences, Beijing 100049, China; ⊥ Department of Applied Biology and Chemical Technology, The Hong Kong Polytechnic University, Hong Hum, Kowloon 999077, Hong Kong; # PolyU Shenzhen Research Institute, PolyU Base Building, No.18 Yuexing Road, Hitech Industrial Park, Nanshan District, Shenzhen 518057, China

## Abstract

miRNA sponges, which could regulate downstream gene expression through competitively binding to overexpressed intracellular miRNAs, have gained increasing attention in miRNA-based therapies. However, linear miRNA sponges typically exhibit low stability and high immunogenicity under physiological conditions. Although artificial circular RNAs (circRNAs) acting as miRNA sponges enhance biostability, immunogenicity still remains a challenge. The 2023 Nobel Prize in Physiology or Medicine highlighted that the incorporation of modified bases could reduce the immunogenicity of exogenous RNAs, yet achieving site-specific modifications in circRNAs remains difficult. Here, we developed a DNA-templated RNA ligation method for the synthesis of site-specifically modified circRNAs with multiple miRNA binding sites, which enabled long-term coregulation of multiple miRNAs. Based on this, we found that 30% pseudouridine modification effectively reduced the immunogenicity of synthetic circular miRNA sponges independent of modification sites or patterns. Taken together, the high yield and site-specific modification of our method enable the precise synthesis of modified circRNAs, which could balance the immunogenicity, biostability, and miRNA binding affinity through engineered modifications in linker regions. These advancements are instrumental for the development of a new generation of circRNA-based therapeutics.

## Graphical abstract

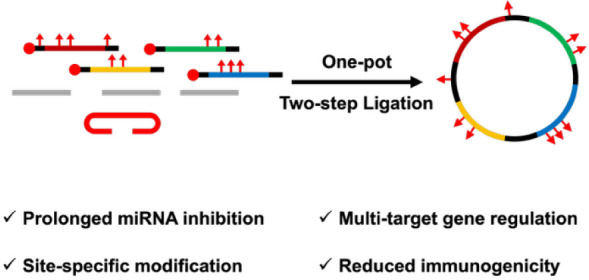

## Introduction

MicroRNAs (miRNAs) are a class of small noncoding RNAs that play an indispensable regulatory role in mRNA degradation, gene expression, and intercellular signaling.^1–3^ Dysregulation of miRNA contributes to various diseases and disorders, including neurodegenerative diseases, cardiovascular diseases, and human malignancies, making them potentially exploitable therapeutic targets.^4–10^ Over the past decades, accumulating evidence has demonstrated that miRNA sponges, which competitively bind to various types and numbers of miRNAs, can function as molecular sponges to negatively regulate miRNA activity by sequestering miRNAs away from their mRNA targets.^11–18^ Generally, long linear RNA sponges with multiple miRNA binding sites were designed for the inhibition of a single kind or several different kinds of miRNAs.^12^ However, the long linear RNA sponges innately possess low biostability and high immunogenicity, which limits their prospects for biomedical applications.^18^ While the usage of circular RNAs (circRNAs) acting as miRNA sponges instead of linear RNAs could improve the biostability, the immunogenicity still remains challenging.^19–21^

The introduction of chemical modifications was a possible solution. Katalin Karikó and Drew Weissman have found the introduction of nucleoside base modifications, e.g., pseudouridine (Ψ) and *N* ^6^-methyladenosine (m^6^A), could abolish the inflammatory responses of exogenous RNAs.^22–24^ Since then, various chemically modified nucleosides, such as 5-methyluridine (m^5^U), 2-thiouridine (s^2^U), and *N* ^1^-methylpseudouridine (m^1^Ψ), have been developed and widely used in mRNA drugs to reduce the immunogenicity and improve the translation.^25–27^ However, these chemical modifications can only be introduced into long linear RNAs in the patterns of full substitution or random substitution, while introducing site-specific modifications into circRNAs is even more challenging due to the inherent limitation of *in vitro* transcription (IVT) and permuted introns-exons (PIE) method.^28–30^ Moreover, the random distribution of chemical modifications may also impair the function of circRNA.^31–33^ For example, the steric hindrance of the methyl group in m^6^A and m^1^Ψ disturbs the base-pairing, which could reduce the binding affinity between the miRNA binding site and corresponding miRNAs.

Although the chemical solid-phase synthesis enables the site-specific modification in short RNAs, there is still a gap between short RNAs and circRNAs that can act as miRNA sponges. Recently, various methods for the cyclization of single-stranded DNA have been developed using circligase or T4 DNA ligase.^34–40^ However, fewer attempts have been made for single-stranded RNA cyclization.

Herein, we reported a facile, one-pot method for generating a site-specifically modified circular miRNA sponge. It was designed with miRNA binding sites for multiple types of miRNAs, enabling the simultaneous sequestration and neutralization of several target miRNAs. Moreover, a site-specific fluorophore was introduced, allowing for visualization of the subcellular distribution of circRNA post-transfection. Furthermore, we demonstrated that regardless of whether the modification pattern is clustered or dispersed, 33% Ψ incorporation can significantly reduce the immunogenicity of exogenous circRNA without impairing miRNA inhibition. Our findings established a programmable platform for generating customizable circRNAs, which not only advances miRNA sponge design but also provides a foundation for precisely investigating and modulating the immunogenicity of circular RNA. This work paves the way for the development of safer, more effective circRNA-based interventions, offering a valuable toolkit for future miRNA regulatory studies and molecular therapeutic applications.

## Results

### Design and Synthesis of CircRNA Based on Template-Direct Ligation

We first developed a facile one-pot method to synthesize large circular RNA from short RNA fragments, which includes a “linear ligation” step and a “circular ligation” step. As illustrated in Figure 1A, several short RNA precursors with 5′-phosphate groups were first ligated to form a long linear RNA intermediate, directed by several DNA templates in the presence of T4 RNA ligase 2 (linear ligation).^41^ Then a linear DNA was added to form a paperclip-shaped assembly, which could further facilitate the intramolecular cyclization of the long linear RNA product obtained from linear ligation with the assistance of Circligase (circular ligation).^37^ Since short RNA precursors are synthesized via solid-phase chemical synthesis, various chemical modifications can be introduced site-specifically. These modifications are retained during cyclization, enabling the construction of circRNA with single-base resolution modifications. Such site-specific modifications may endow circRNA with distinct and specialized functionalities, thus creating new possibilities for its applications in both biochemical and medical research.

**Figure 1:**
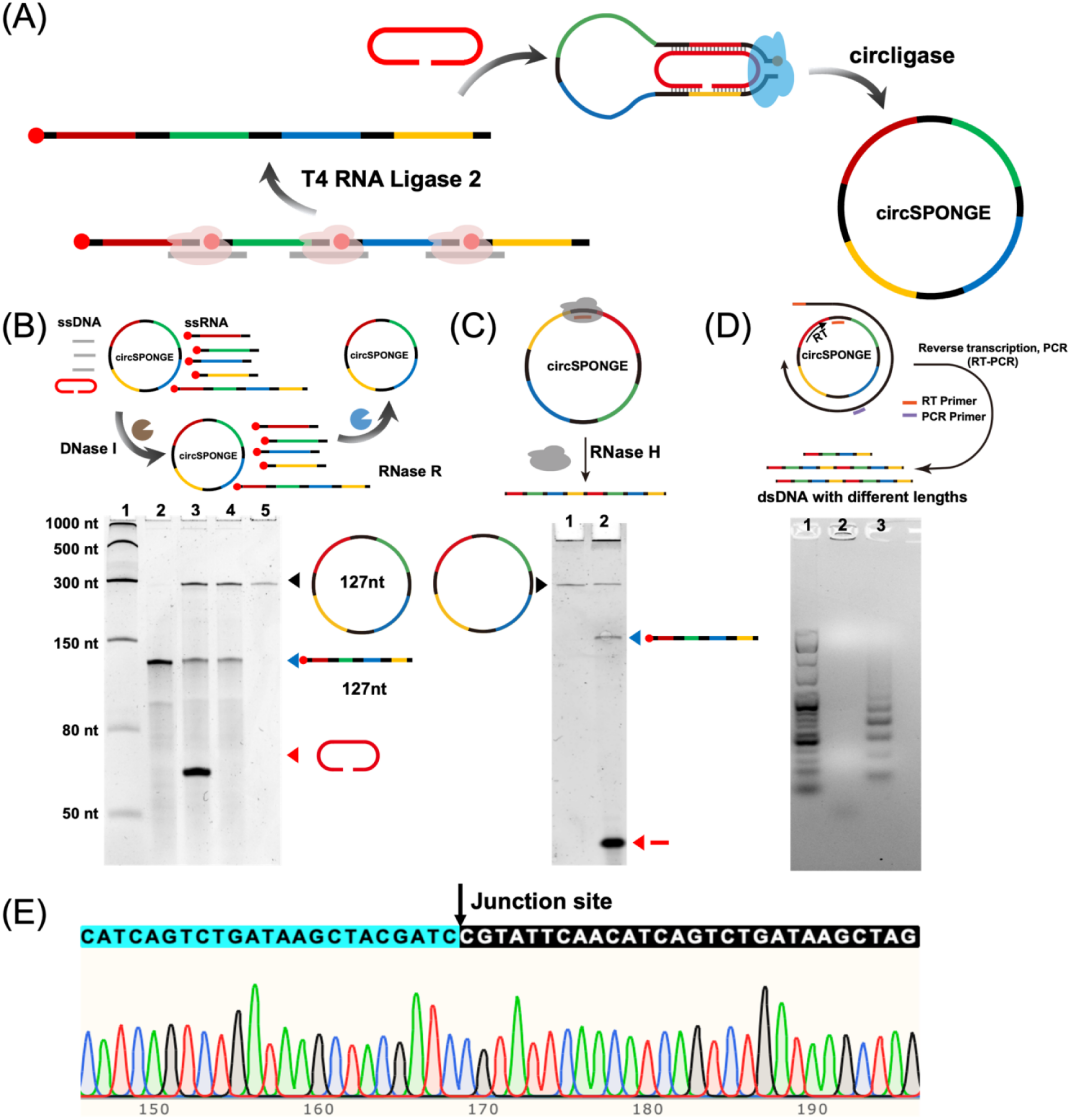
Design and synthesis of circSPONGE. (A) Synthesis strategy of circSPONGE. (B) Characterization of the synthesis and purification of circSPONGE through 10% denaturing PAGE. Lane 1–5: low-range ssRNA ladder; linear ligation; circular ligation; DNase I digestion product; RNase R digestion product. (C) The characterization of the RNase H-mediated cleavage of circSPONGE. Lane 1 and 2: circSPONGE; RNase H cleavage product. (D) The reverse transcription and PCR products were characterized through 1% agarose gel electrophoresis. Lane 1–3: dsDNA marker; reverse transcription product; and PCR product. (E) Sanger sequencing results of the PCR product.

In a typical experiment, a 127 nt-long circRNA (circSPONGE, sequences are shown in Table S1) was designed (Scheme 1). This construct incorporates four miRNA binding sites: two for miR-21, one for miR-221, and one for miR-195. These miRNAs act as key regulators driving tumor proliferation and invasion, and they are frequently upregulated in cancers such as breast and liver cancer.^42–44^ To alleviate the potential interference between multiple miRNA binding sites, a ten-nucleotide linker was introduced between each miRNA binding site. The linker sequence was carefully designed through the predicted secondary structure to avoid the strong interaction between linker regions and miRNA binding sites (Figure S1). As shown in Figure 1B, after “linear ligation”, a new band (lane 2) with slower migration appeared, which indicated the generation of a 127 nt-long linear RNA intermediate (linSPONGE) through the ligation of short RNA precursors. The yield of the linear ligation was quantified to be approximately 95% through grayscale analysis. After the second “circular ligation” step, a distinct new band with slower migration was observed as depicted in lane 3, which was resistant to RNase R digestion (lane 5), suggesting a circular structure. Since the composition of the assembly buffer plays a critical role in the hybridization between DNA templates and short RNA precursors, as well as the overall yield of circRNA, several commonly used buffer conditions were tested. As shown in Figure S2, buffers containing Mg^2+^ or Ca^2+^ achieved cyclization efficiencies of approximately 40–50% while being lower in the presence of Na^+^ or K^+^. This difference may be attributed to the ability of divalent ions, such as Mg^2+^ and Ca^2+^, to stabilize DNA/RNA hybrids and enhance the ligase activity. The monomeric circular product was further confirmed by MALDI-TOF, with a molecular weight of 40544 g/mol (Figure S3), consistent with the theoretical molecular weight of 40544 g/mol. Moreover, the purification of circRNA can be effectively accomplished through sequential treatment with DNase I and RNase R, enabling the removal of DNA templates and single-stranded RNA components, respectively.

**Scheme 1:**
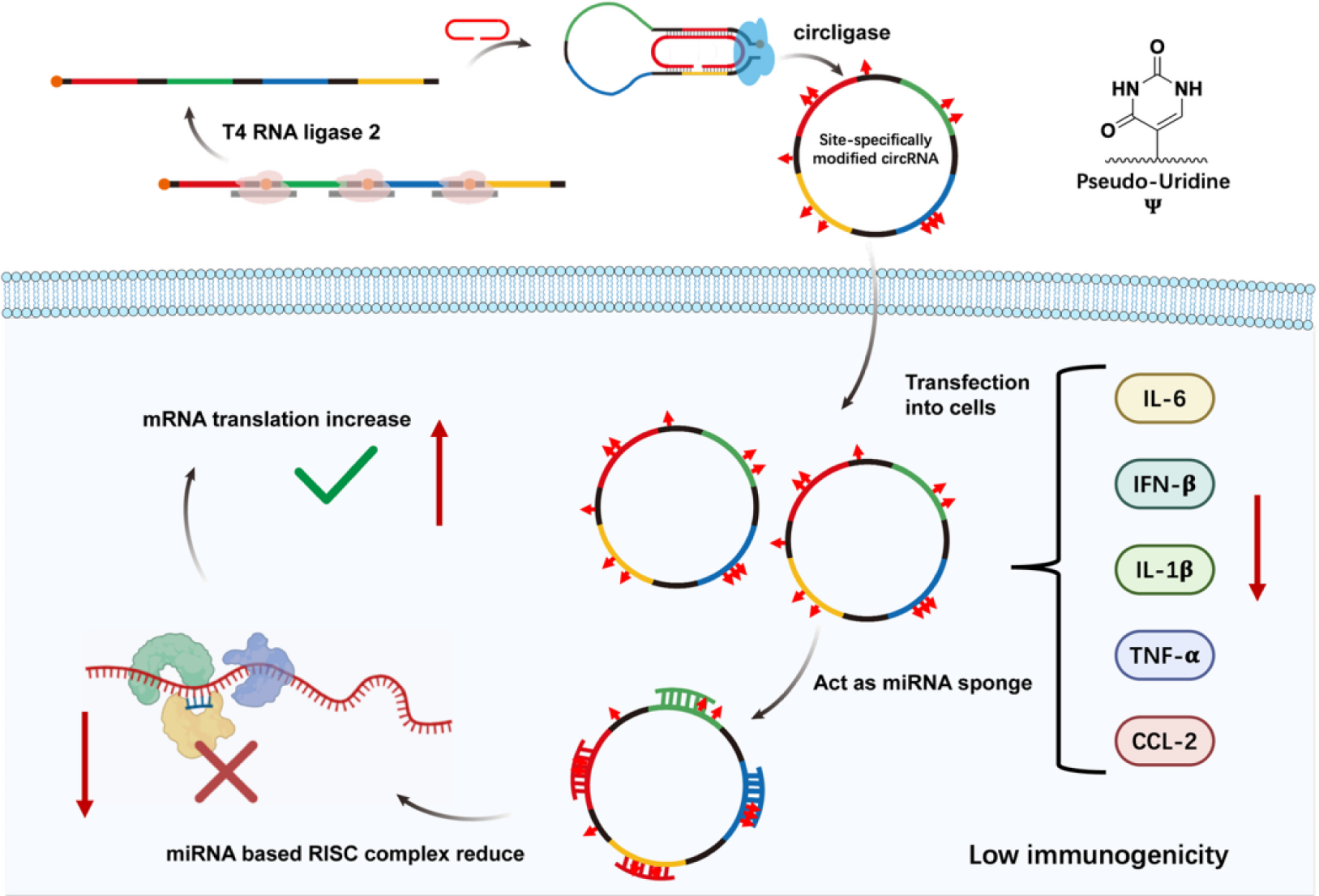
Synthesis of Site-Specifically Modified Circular miRNA Sponge with Low Immunogenicity for Simultaneous Multiple miRNAs Inhibition

To further validate the circular structure of the final product, RNase H-mediated cleavage was also employed, as this enzyme specifically hydrolyzes the RNA segment in RNA/DNA hybrids. As shown in Figure 1C, after annealing with a complementary DNA strand, the RNase H treatment generated a distinct band with an increased migration rate. Since this DNA strand was complementary to the junction site, this result indicated that the final product had a circular structure. In addition, we investigated the circular structure via reverse transcription PCR (RT-PCR) using divergent primers followed by Sanger sequencing. As illustrated in Figure 1D, agarose gel electrophoresis of the RT-PCR products revealed multiple bands after RT-PCR, indicating the presence of circular RNA species. The sequencing results (Figure 1E) further confirmed the formation of a phosphodiester bond at the junction site, a hallmark of circRNA. Taken together, these results validated both the structural integrity and sequence fidelity of the final circRNA obtained through our DNA-templated RNA ligation approach.

### Silence of Multiple MicroRNAs in MCF-7 Cells

The simultaneous binding capability of the three selected miRNAs was first verified through an *in vitro* assembly assay. As shown in Figure S4, the bands in Lanes 5 to 7 suggested that the covalently closed circular structure of circSPONGE did not affect the binding of individual miRNAs. Furthermore, the band in Lane 8 indicated that all three different miRNAs can bind to a single circSPONGE molecule concurrently. To further validate the multitarget regulatory function of circSPONGE, which contains four miRNA binding sites, we employed a dual-luciferase reporter system to evaluate miRNA activity. Taking the miR-21 reporter as an example, the miR-21 binding site was inserted into the 3′-untranslated region (3′-UTR) of the firefly luciferase gene. Through its decoy effect, circSPONGE sequesters miR-21, thereby disrupting the miR-21-induced silencing complex and increasing firefly luciferase expression. Meanwhile, Renilla luciferase served as an internal control, unaffected by miRNA levels, enabling normalized quantification of regulatory efficacy through the ratio of firefly to Renilla luciferase activity (normalized firefly activity, NFA). As shown in Figure 2B, circSPONGE treatments significantly increased NFA values (2.5, 3.3, and 6.3) compared to the cmiRNA mix (a mixture of linear AMOs including cmiR-21, cmiR-221, and cmiR-195 at a 2:1:1 ratio), whereas no such upregulation was observed in the circScr group, a circRNA of identical length to circSPONGE but lacking miRNA binding sites (sequence shown in Table S2). This result indicated that circSPONGE exhibits higher potency than linear AMO in the sequestration of miR-21. Moreover, this sequestration occurred in a dose-dependent manner, with increasing concentrations (10 nM, 20 nM, 30 nM) resulting in higher NFA values, making it highly desirable for both research and clinical applications.

**Figure 2:**
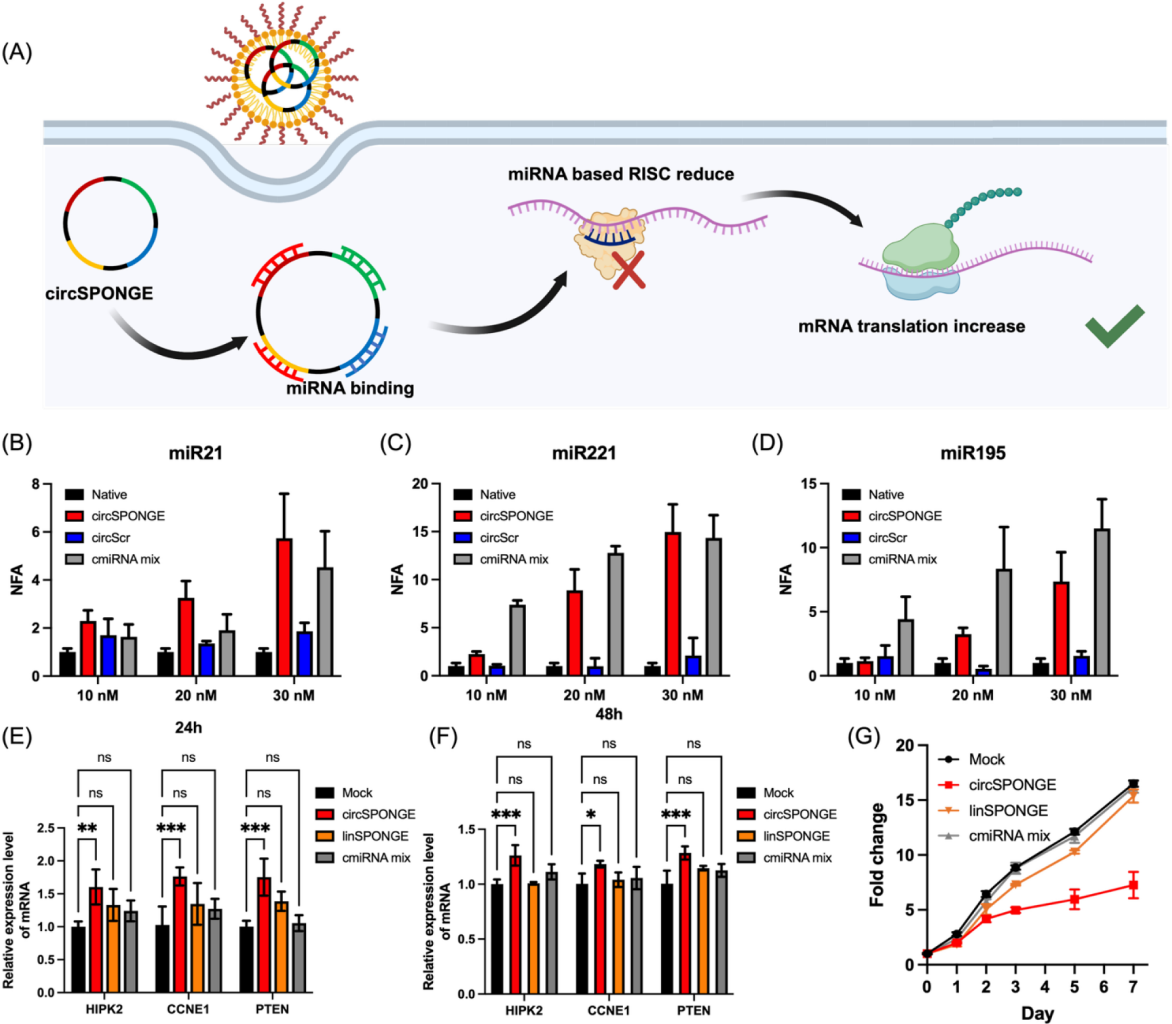
circSPONGE decoys multiple miRNAs in MCF-7 cells. (A) Schematic illustration of the simultaneous inhibition of multiple miRNAs by using circSPONGE. (B–D) Dual-luciferase reporter results for miR-21 (B), miR-221 (C), and miR-195 (D). (E–F) Relative expression levels of downstream mRNAs at 24 h (E) and 48 h (F) after transfection with 30 nM miRNA inhibitors (circSPONGE, linSPONGE, and cmiRNA mix). The mRNA expression levels were measured through RT-qPCR using GAPDH mRNA as an internal control; at least three independent experiments were performed, and multiple comparisons were performed using one-way ANOVA with Tukey’s test. *P < 0.05, **P < 0.01, ***P < 0.001, and ****P < 0.0001. (G) Proliferation inhibition of MCF-7 cells under circSPONGE, linSPONGE and cmiRNA mix treatments.

The designed circSPONGE also contains two additional sites complementary to miR-221 and miR-195, respectively. We further evaluated the sequestration effect of circSPONGE on miR-221 and miR-195 using dual-luciferase assays. As shown in Figure 2C and D, results similar to those for miR-21 indicated that circSPONGE treatments increased NFA values, demonstrating the successful sequestration of the target miRNAs and subsequent restoration of firefly luciferase expression. However, circSPONGE showed lower NFA values compared to the corresponding cmiRNA mix in miR-221 and miR-195 reporter assays. For instance, in the experiment for miR-221 at 10 nM, the normalized NFA values for circSPONGE and cmiRNA mix were 2.8 and 4.9, respectively. We hypothesized that this discrepancy in NFA values between circSPONGE and cmiRNA mix likely arises from the varying number of binding sites within circSPONGE, with two binding sites for miR-21 but only one each for miR-221 and miR-195. Consequently, the relatively higher effective concentration and synergistic effects of the two binding regions contributed to the superior miRNA adsorption capability of circSPONGE for miR-21 compared to miR-221 and miR-195. To verify the correlation between the number of miRNA binding sites and sponge potency, we engineered a variant circSPONGE containing two miRNA binding sites for miR-221, alongside single sites for miR-21 and miR-195. As shown in Figure S5B–D, increasing the miR-221 binding sites resulted in a marked improvement in inhibitory efficiency toward miR-221, achieving levels comparable to those observed for miR-21 in the original dual-site design (Figure 2B). These findings demonstrated that increasing the number of miRNA binding sites effectively enhances the miRNA suppression capacity of the sponge. Taken together, these results demonstrated that circSPONGE could act as a multitargeted miRNA sponge, effectively sequestering various miRNAs simultaneously. In particular, the enhanced miRNA sequestration effect of circSPONGE could be attributed to the synergistic enhancement provided by multiple binding sites.

After characterizing the inhibition of the miRNA-induced silencing complex, we evaluated the relative expression levels of downstream mRNAs at different time points post-transfection through RT-qPCR. Three mRNAs were selected: phosphatase and tensin homolog (PTEN, a target for miR-21), homeodomain-interacting protein kinase 2 (HIPK2, a target for miR-221), and cyclin E1 (CCNE1, a target for miR-195). As depicted in Figure 2E, at 24 h post-transfection, circSPONGE significantly increased the mRNA expression levels of HIPK2, CCNE1, and PTEN by 50%, 76%, and 75%, respectively, whereas linSPONGE and cmiRNA mix exhibited marginally increased expression levels. Similar results were observed at 12h (Figure S6A) and 36h (Figure S6B) post-transfection. Notably, at 48 h post-transfection (Figure 2F), there was no significant difference in mRNA expression levels between the cmiRNA mix group and the mock group, whereas circSPONGE still significantly upregulated the mRNA expression levels. These results indicated that circSPONGE was more effective in inhibiting the corresponding miRNAs and activating the expression of downstream mRNAs (HIPK2, CCNE1, and PTEN) compared to the corresponding linear RNAs, especially over long time scales. We hypothesized that the enhanced stability of circSPONGE, which arises from the absence of terminal ends, contributes to its superior gene upregulation effect. The biostability of circSPONGE was further evaluated through incubation with RNase R and fetal bovine serum (FBS). As shown in Figure S7, circSPONGE remained intact after incubation with RNase R for 24 h, while linSPONGE was completely degraded after just 1 h. Furthermore, in the presence of 10% FBS, ∼40% circSPONGE remained intact even after 48 h incubation, whereas linSPONGE was fully degraded after 1 h. Hence, the covalently closed circular structure endowed circSPONGE with exceptional stability, facilitating long-term multitargeted miRNA inhibition and downstream mRNA regulation.

The synergistic impact of this multitargeted miRNA regulation on cell proliferation was further assessed. As shown in Figure 2G, linSPONGE exhibited only slight inhibition of cell proliferation, while circSPONGE significantly suppressed the cell proliferation even after 7 days. This sustained efficacy likely stems from the enhanced biostability and multitarget miRNA regulatory capacity of circSPONGE.

### Synthesis of Site-Specifically Fluorophores Modified CircRNA

In our synthetic approach, short RNA precursors are obtained through chemical solid-phase phosphoramidite synthesis. This enables the introduction of various chemical modifications with single-base resolution, which are subsequently retained in the final circRNA product. As a proof of principle, we selected three distinct fluorophores to design and synthesize circRNA featuring triple-fluorescent modifications. As shown in Figure 3A, three noninterfering fluorescent modifications (FAM, Cy3, and Cy5) were incorporated into three different short RNA precursors (sequences were shown in Table S5). Following the two-step ligation process described earlier, these modifications were simultaneously and site-specifically integrated into circRNA. By overlapping the fluorescence scans from the FAM, Cy3, and Cy5 channels in PAGE analysis, we confirmed the presence of all three modifications in both the long linear RNA intermediate and the final circRNA product. These results demonstrate that our method is compatible with the synthesis of site-specifically modified circRNAs.

**Figure 3:**
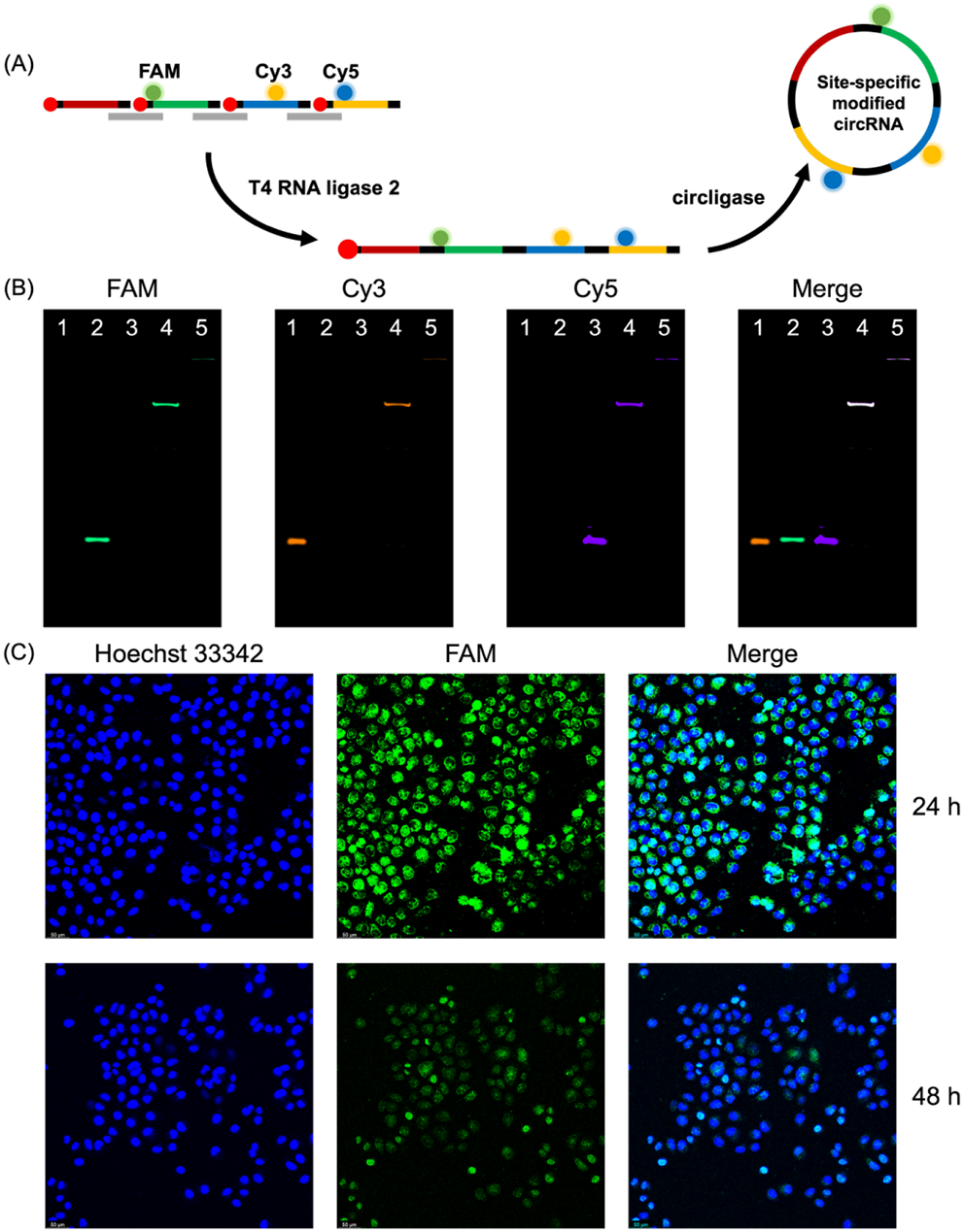
Synthesis of site-specifically fluorophore modified circSPONGE and investigation of the subcellular distribution of circSPONGE. (A) The synthesis route of site-specifically fluorophores modified circSPONGE. (B) 10% denaturing PAGE analysis of the synthesis and purification of site-specifically fluorophores modified circRNA. Lane 1–5: Cy3-cirmir-195; FAM-cirmir-221; Cy5-cirmir-21B; linear ligation; circular ligation. (C) Confocal images of MCF-7 cells transfected with 30 nM FAM-modified circRNA 24 and 48 h post-transfection. The nucleus was stained with Hoechst 33342.

Furthermore, we designed and synthesized FAM-labeled circSPONGE (Figure S8) to investigate its subcellular distribution following transfection into MCF-7 cells. As shown in Figure 3C, at 24 and 48 h post-transfection, circSPONGE was observed in both the nucleus and cytoplasm. Since the miRNA-induced silencing complex is primarily localized in the cytoplasm, this distribution pattern is consistent with the gene-regulatory function of circSPONGE. In summary, our synthetic method enables the introduction of chemical modifications with single-base precision at specific sites, which holds great significance for functional studies on site-specific chemical modifications of circRNAs.

### Site-Specifically Pseudouridine Modification Reduced the Immunogenicity of CircRNA

Although circular RNAs (circRNAs) were initially reported to exhibit reduced immunogenicity compared to linear RNAs, and certain highly purified circRNAs evade immune detection,^45^ emerging evidence reveals that synthetic circRNAs can still provoke innate immune responses due to secondary structures or sequence motifs inadvertently mimicking viral pathogen-associated molecular patterns (PAMPs).^19^ To abrogate this innate cellular immune response, nucleoside modifications such as pseudouridine (Ψ), *N* ^1^-methylpseudouridine (m^1^Ψ), and 5-methoxyuridine (5moU) have been developed for linear mRNA, which can also be adapted for circRNAs. However, current approaches for circRNA modification are constrained by random or complete uridine substitutions (e.g., replacing all uridines with Ψ), lacking precision in site-specific engineering. In this study, a series of site-specifically Ψ-modified circRNAs (sequences were shown in Table S6) with different densities and patterns were designed and synthesized to elucidate how the ratio and position of nucleoside modifications affect the immunogenicity of exogenous circRNA. These modified circRNAs were synthesized based on our innovative short-strand ligation strategy. Specifically, the 5′-phosphorylated RNA short strands with precise Ψ modifications were prepared through solid-phase synthesis (Figure S9), which were then ligated to prepare the site-specific Ψ-modified circRNAs (Figure S10). The modification patterns are shown in Figure 4A. Given that circSPONGE contains 33 uridines, we first designed circ-33 Ψ, in which all uridines were replaced with Ψ. Additionally, we developed two different modification patterns, a clustered pattern (circ-10 Ψ) and a randomly dispersed pattern (circ-11 Ψ), to investigate the influence of modification position.

**Figure 4:**
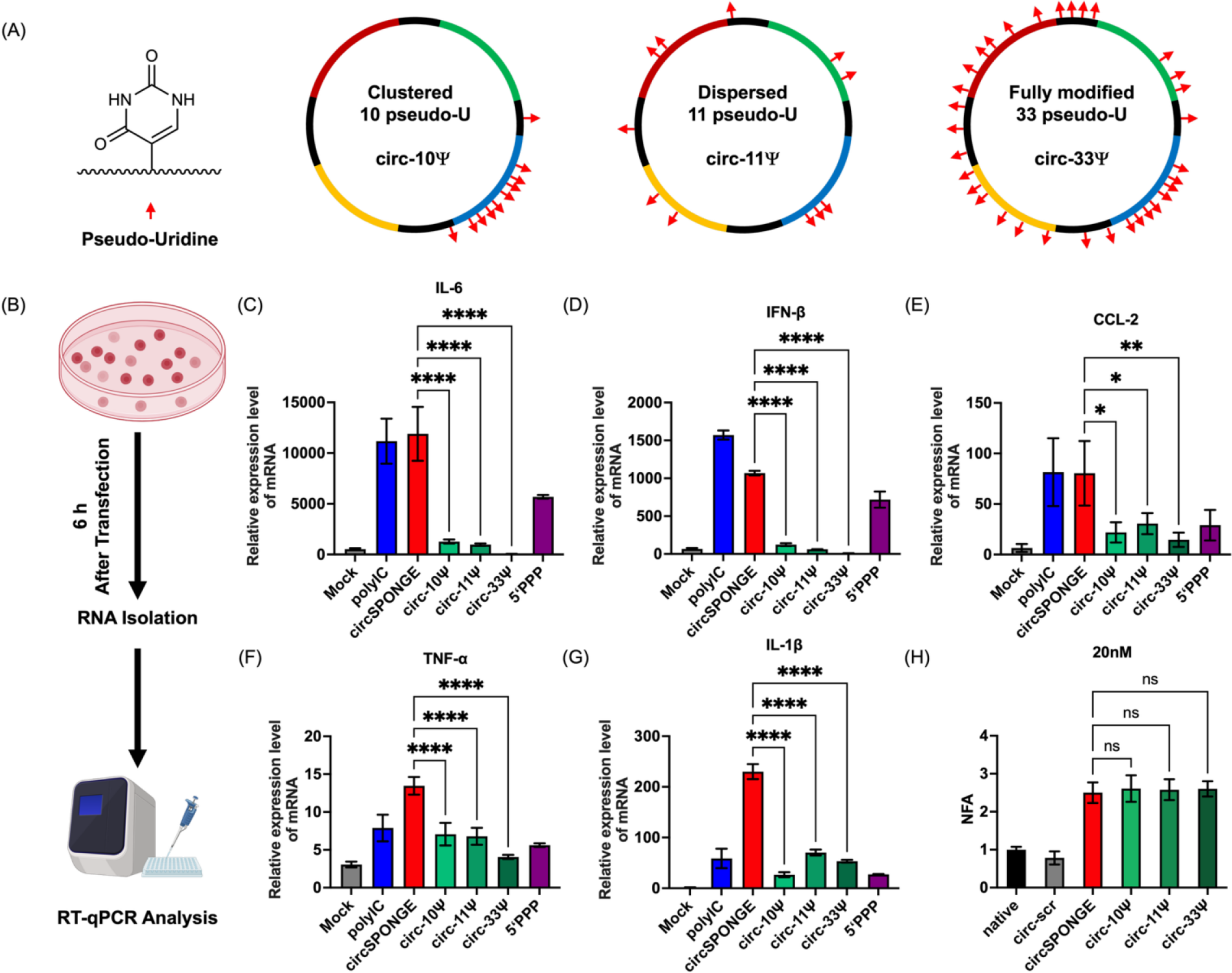
Site-specific pseudouridine modifications attenuate the immunogenicity without compromising miRNA silencing. (A) Chemical structure of pseudouridine and its distribution patterns in circ-10 Ψ, circ-11 Ψ, and circ-33 Ψ. (B) Assessment of immunogenicity for circRNAs with varying pseudouridine-modified patterns in RAW 264.7 cells. (C–G) The relative mRNA expression of IL-6 (C), IFN-β (D), CCL-2 (E), TNF-α (F), and IL-1β (G) in RAW 264.7 cells after transfection with 20 nM circSPONGE. (H) Dual-luciferase reporter results for miR-21 after transfection with 20 nM circSPONGE. The mRNA expression was measured through RT-qPCR using GAPDH as the internal control. Data represent at least three independent experiments, with multiple comparisons performed using one-way ANOVA followed by Tukey’s test. *P < 0.05, **P < 0.01, ***P < 0.001, and ****P < 0.0001.

To investigate the immunogenicity of the designed circRNAs, we selected a murine stable macrophage cell line (RAW264.7). As shown in Figure 4B, the evaluation of the innate cellular immune response was achieved by quantifying mRNA expression levels of several key proinflammatory cytokines and chemokines, including secreted interferon-β (IFN-β), interleukin-6 (IL-6), chemokine (C–C motif) ligand 2 (CCL-2), tumor necrosis factor-α (TNF-α) and interleukin 1β (IL-1β). As illustrated in Figure 4C–G, the expression levels of these cytokines and chemokines were upregulated upon transfection with poly­(I:C), unmodified circRNA, or 5′-triphosphate linear RNA (synthesized via IVT). Notably, the incorporation of Ψ significantly attenuated the immunogenicity of circRNA: compared with unmodified circRNA, transfection with Ψ-modified circRNAs at different modification levels (circ-10Ψ, circ-11Ψ, and circ-33Ψ) led to a substantial reduction in cytokine expression. Importantly, full Ψ modification (circ-33Ψ) induced an immunogenic response characterized by virtually undetectable levels of secreted cytokines. Remarkably, a 30% modification rate (circ-10Ψ) was sufficient to achieve immunogenicity suppression comparable to that of full modification (circ-33Ψ). Furthermore, the two distinct modification patterns (circ-10Ψ and circ-11Ψ) exhibited similar efficacy in abrogating the immune response. These findings underscore that the quantity of pseudouridine modifications is the primary determinant of circRNA immunogenicity reduction, whereas the specific positions of these modifications have a negligible impact on this effect. This insight paves the way for optimized nucleoside modification strategies that minimize immune activation while preserving the therapeutic efficacy.

Since Ψ and uridine (U) exhibit similar base-pairing properties, this modification is expected to have a minimal impact on the hybridization between circSPONGE and its target miRNAs. To confirm this, we performed dual-luciferase reporter assays. As illustrated in Figure 4H, at a concentration of 20 nM, the normalized firefly activity (NFA) values of circ-10Ψ, circ-11Ψ, circ-33Ψ, and unmodified circRNA showed no significant differences. This indicates that the Ψ modification ratio and pattern reduce the immunogenicity of exogenous circRNA without compromising its miRNA-sponging activity.

## Conclusion

In conclusion, we report a facile one-pot method for synthesizing site-specifically modified circular RNA, enabling the precise incorporation of chemical modifications at single-base resolution. This approach overcomes the limitations of traditional IVT and PIE methods, which often produce immunogenic byproducts or lack precise control over modification sites. By designing and synthesizing circSPONGE with multiple miRNA binding sites for three oncogenic miRNAs (miR-21, miR-221, and miR-195), we demonstrated its superior efficacy as a miRNA decoy compared to linear anti-miRNA oligonucleotides (AMOs).

A primary advantage of circRNA sponges lies in their role as natural, stable miRNA decoys. Unlike linear AMOs, which suffer from short half-lives, circRNAs exhibit an enhanced resistance to exonuclease degradation, prolonging their circulation time and therapeutic persistence. Our results showed that circSPONGE effectively sequesters multiple miRNAs simultaneously and in a dose-dependent manner, as evidenced by dual-luciferase reporter assay and RT-qPCR results. This multitarget sequestration capability—achieved through the incorporation of multiple binding sites—outperforms equimolar mixtures of linear AMOs by synergistically downregulating oncogenic miRNAs, thereby effectively suppressing MCF-7 cell proliferation. Such simultaneous neutralization of diverse miRNAs is particularly advantageous for treating complex diseases involving miRNA dysregulation networks such as cancers, neurodegenerative disorders, and cardiovascular conditions, where single-target therapies often fall short.

Moreover, fluorescently modified circRNA was synthesized to demonstrate the compatibility of our synthetic method with site-specific modifications. Building on this, the introduction of site-specific Ψ modifications significantly suppresses the immunogenicity of circRNA and decreases the expression of multiple proinflammatory cytokines without compromising its miRNA-sponging function. Transfection experiments in RAW264.7 cells revealed that even a 30% Ψ modification ratio effectively abrogated innate immune responses, minimizing proinflammatory cytokine secretion (e.g., IFN-β, IL-6, TNF-α) to levels comparable to full substitution, far superior to unmodified circRNA. This site-specific control avoids the drawbacks of random or excessive modifications, which can disrupt base-pairing and impair functionality, while enabling additional features like fluorophore labeling for subcellular visualization.

In summary, this approach provides a facile and efficient platform for generating customized circRNAs with tailored modifications. It not only advances the development of miRNA sponge therapeutics but also establishes a foundation for investigating the origins of immunogenicity and precisely modulating immunogenicity in exogenous RNA, which is beneficial for the development of novel tools for gene regulation research and molecular therapeutic applications. Future work could explore *in vivo* delivery systems and clinical translation, paving the way for safer and more effective circRNA-based miRNA interventions in precision medicine.

## Supplementary Material



## Notes

Experimental details, materials, methods, sequence information, and some experimental results, including the MALDI-TOF-MS, optimization of reaction condition, PAGE, and so on (PDF)
