# Supplementary material for: Site-Specifically Modified Circular Ribonucleic Acid Serves as Multitarget miRNA Sponge with Low Immunogenicity
Source: J Am Chem Soc. 2026 Apr 11;148(15):16493–501. doi: 10.1021/jacs.6c04991 (PMC13107438; doi:10.1021/jacs.6c04991)
Supplement: Supplementary file 1 [file ja6c04991_si_001.pdf]

Supporting information

Site-Specifically Modified Circular Ribonucleic Acid Serves as Multi-Target miRNA Sponge with Low Immunogenicity

Yufan Pan[1, $], Xin Li[1, $], Bini Zhou[1], Yuan Zhuang[1], Liangzhi Luo[2], Chenyou Zhu[1], Rui Xu[1], Yifan Jiang[1], Yuanchen Dong[3,4, *], Ziyang Hao[2, *], Dongsheng Liu[1,5,6 *], Xi Zhang[1]

AUTHOR ADDRESS

[1] Department of Chemistry, Tsinghua University, Beijing, 100084, China

[2] School of Pharmaceutical Sciences, Capital Medical University, Beijing, 100069, China

[3] CAS Key Laboratory of Colloid Interface and Chemical Thermodynamics, Beijing National Laboratory for Molecular Sciences, Institute of Chemistry, Chinese Academy of Sciences, Beijing, 100190, China

[4] University of Chinese Academy of Sciences, Beijing, 100049, China

[5] Department of Applied Biology and Chemical Technology, The Hong Kong Polytechnic University, Hong Hum, Kowloon, 999077, Hong Kong

[6] PolyU Shenzhen Research Institute, PolyU Base Building, No.18 Yuexing Road, Hitech Industrial Park, Nanshan District, Shenzhen, 518057, China

Materials and Methods

The DNA templates and 5-phosphate modified short RNA precursors were ordered from Suzhou Biosyntech Co., Ltd. Circleligase II and RNase R was purchased from novoprotein. T4 RNA ligase 2, RNase H, DNase I, PmeI and XbaI restriction endonucleases were acquired from New England Biolabs. RevertAid First Strand cDNA Synthesis Kit and Phusion Plus PCR Master Mix were bought from Thermo Fisher Scientific. pmirGLO Dual-Luciferase miRNA Target Expression Vector, Dual-Luciferase® Reporter Assay System and CellTiter 96® AQ_{ueous} One Solution Cell Proliferation Assay were purchased from Promega. Lipofectamine® MessengerMAX™ was acquired from Invitrogen. Dulbecco's modified eagle medium (DMEM), Opti-MEM™ I Reduced Serum Medium and fetal bovine serum (FBS) were purchased from Gibco. FastPure Cell/Tissue Total RNA Isolation Kit V2 was purchased from Vazyme. M5 Super plus qPCR RT kit with gDNA remover and 2× M5 HiPer SYBR Premix EsTaq (with Tli RNaseH) were purchased from Mei5bio. Hoechst 33342 was purchased from LabLead company.

1. **Synthesis of circular RNA**

   The 5-phosphate modified short RNA precursors were mixed with corresponding DNA templates at a molar ratio of 1: 1 in 1×TAE-Mg^{2+}(12.5 mM) at 10 uM. Then the mixture was incubated at 75°C for 5 min and then cool to 30°C at a rate of 0.5°C per minute. After that, T4 RNA Ligase 2 (NEB, M0239L) and ligase buffer were added and the mixture were incubated at 37°C for 4h for linear ligation. After the reaction, the mixture is subjected to ultrafiltration using an ultrafiltration tube with a molecular weight cutoff of 3 kDa to remove salts and concentrate the reaction mixture through ultracentrifugation to obtain the linear long RNA intermediate.

   The concentration of linear intermediates was determined using Nanodrop (Thermo Fisher Scientific, USA) and an equimolar amount of cycle strand was added at a concentration of 1 umol/L in 1× TAE-Mg^{2+} (12.5 mmol/L). The mixture is then heated to 75 °C, incubated for 5 minutes, and gradually cooled to 4°C at a rate of 0.5°C per minute. The annealed circular RNA assembly are mixed with 10× circligase reaction buffer and 50 mM $MnCl_2$ to a final concentration of 0.5 μmol/L. Then Circleligase II (Novoprotein, M027) was added, and the mixture was kept at 45 °C for 2 hours for the circular ligation.

2. **Purification of circular RNA**

   For the removement of the DNA templates, 5 uL DNase I (NEB, M0303L) was added to 1 nmol of the reaction mixture after circular ligation and the mixture was then incubation at 37°C for 1h for the digestion of all of the DNA. After that, 0.5 uL RNase R (novoprotein, E224-01A) was added to 1 nmol of the product after DNase I digestion and the mixture was incubated at 37°C for 30 min to remove all of the linear RNA. Then the final product was ethanol precipitated and dissolved in DEPC H_2O.

3. **RNase H mediated cleavage of circular RNA**

10 pmol synthesized circSPONGE was annealed with a DNA strand which is complementary to the 20 nt around junction site at 1×PBS buffer with the molar ratio of 1: 1. Then 1 uL RNase H (NEB, M0297S) was added and the mixture was incubated at 37°C for 30 min. The reaction mixture was then incubated at 65°C for 30 min to stop the reaction and the cleaved product was analyzed through 10% denaturing PAGE.

4. **Reverse transcription and PCA amplification of circular RNA**

250 ng synthesized circSPONGE was reverse transcribed using DNA-C1 as primer through RevertAid First Strand cDNA Synthesis Kit (Thermo Scientific™, K1622). Then the cDNA was amplified through Phusion Plus PCR Master Mix (Thermo Scientific™, F632S).

5. **Binding of circSPONGE and miRNAs**

The circSPONGE was mixed with miR-21 at a molar ratio of 1:2 and miR-221/ miR-195 at a molar ration of 1:1 in 1×PBS at 2 uM final concentration. The mixture is then heated to 75 °C, incubated for 5 minutes, and gradually cooled to 4°C at a rate of 0.5°C per minute.

6. **Dual-luciferase report assay**

MCF-7 cell lines were seeded into a 96-well plate at a density of 8×10^4 cells/mL, with 100 μL of culture medium (DMEM containing 10% FBS) per well. After overnight incubation, the medium was replaced with fresh culture medium, 100 ng reporter plasmid and various amounts of RNA (circSPONGE, circScr, cmiRNA mix) was transfected using Lipofectamine® MessengerMAX™ (Invitrogen™, LMRNA015) according to the manufacturer's instructions. The cells continued to culture under conditions of 37°C and 5% CO_2. 24 hours after transfection, the Dual-Luciferase® Reporter Assay System (Promega, E1910) was used for the evaluation of Fluc and Rluc activity. The relative expression level of Fluc to Rluc was calculated and normalized to native group using GraphPad Prism 9.

7. **Total RNA isolation and RT-qPCR analysis**

For the evaluation of the downstream mRNA expression level, MCF-7 cell lines were seeded into a 12-well plate at a density of 4×10^5 cells/mL, with 1 mL culture medium. Then 30 nM circSPONGE, circScr or cmiRNA mix were transfected after 24h through Lipofectamine® MessengerMAX™ (Invitrogen™, LMRNA015). The cells were harvested at 12h, 24h, 36h and 48h after transfection.

For the evaluation of immunogenicity related mRNA expression level, RAW 264.7 cells were seeded into a 12-well plate at a density of 3×10^5 cells/mL, with 1 mL culture medium. Then 1 ug RNA was transfected using Lipofectamine® MessengerMAX™ (Invitrogen™, LMRNA015). The cells were harvested 6h after transfection.

The total RNA was isolated using FastPure Cell/Tissue Total RNA Isolation Kit V2 (Vazyme, RC112-01) according to the manufacturer's instructions. Then 1 μg total RNA was used for cDNA synthesis using M5 Super plus qPCR RT kit with gDNA remover (Mei5bio, MF166-plus-01). The real-time PCR was performed using 2X M5 HiPer SYBR Premix EsTaq (with Tli RNaseH) (Mei5bio, MF787-NR-01) according to the manufacturer's instructions through LightCycle96

(Roche). The relative expression levels of mRNAs were normalized to GAPDH mRNA and analysis using GraphPad Prism 9. The primer sequences for qPCRwere listed is Table S4.

8. **Biostability evaluation**

For the stability test in the present of exonuclease, 2ug RNA (linSPONGE or circSPONGE) was incubated with 2U RNase R (novoprotein, E224-01A) in a 50 uL reaction mixture at 37°C. Samples were collected at different timepoints and the Rnase R was inactivated by incubation at 70°C at 5 min according to the manufacturer's instructions.

For the stability test in the present of FBS, 2ug RNA (linSPONGE or circSPONGE) was incubated with 5uL FBS in a 50 uL reaction mixture at 37°C. Samples were collected at different timepoints mixed with formamide immediately, then the samples were heated to 75°C for 5 min to denature nucleases.

9. **Evaluation of cell proliferation**

Human breast cancer cell line MCF-7 was seeded into a 96-well plate at a density of 5×10^4 cells/mL, with 100 μL of culture medium added per well. After overnight incubation, the medium was replaced with fresh medium, and various amounts of RNA (circASPONGE or cmiRNA mix) was transfected into the cells in the wells according to the experimental design using Lipofectamine® MessengerMAX™ (Invitrogen™, LMRNA015), with a final concentration of 30 nM. After gentle mixing, the cells were further incubated under conditions of 37°C and 5% CO_2. Cell viability was assessed at 24 h, 48 h, 72 h, and 120 h post-transfection using CellTiter 96® AQ_{ueous} One Solution Cell Proliferation Assay (Promega, G3580). Briefly, 10 μL of MTS reagent was added to each well and returned to the incubator for 1 h. The absorbance of the samples at 450 nm and 690 nm was then measured using a plate reader. Data were processed using Excel and GraphPad Prism 9 software, with the difference in absorbance values at the two wavelengths used to characterize the level of cell viability.

10. **Subcellular distribution of FAM label circular RNA**

MCF-7 cells were seeded into a 96-well plate at a density of 5×10^4 cells/mL, with 100 μL of culture medium (DMEM containing 10% FBS) per well. After overnight incubation, 3 pmol FAM labelled circSPONGE was transfected using Lipofectamine® MessengerMAX™ (Invitrogen™, LMRNA015) according to the manufacturer's instructions per well. The cells continued to culture under conditions of 37°C and 5% CO_2. 24h and 48h post-transfection, the cell nuclei were stained using a prepared Hoechst 33342 (LabLead, B2662) staining solution. The staining solution was prepared by diluting 400 μL of a 1 mM Hoechst 33342 stock solution into 600 μL of culture medium. The medium in the 96-well plate was removed, and 100 μL of the staining solution was added to each well. After staining for 10 min, the staining solution was removed and the cells were washed with PBS for three times. Then the plate was placed in a fluorescence confocal microscope under excitation wavelength of 488 nm and 405 nm for FAM and Hoechst 33342, respectively.

11. **Synthesis of pseudo-uridine modified circRNA**

The pseudo-uridine modified RNA precursors were synthesized by a HY12 oligo synthesizer (Highgene Automation) using commercially available 2'-OTBDMS protected ribonucleoside phosphoramidites (100 mM in CH_3CN) on controlled pore glass support (1 μmol scale). After synthesis, the RNA loaded CPG was put in 1 mL concentrated ammonium hydroxide and heated at 60 °C for 3 h to cleave the RNA from CPG. Then the supernatant was concentrated to dryness and 100 μL of DMSO and 125 μL of $NEt_3 \cdot 3HF$ were added and the mixture was heated at 65°C for 2 hours to remove the TBDMS protecting group. Subsequently, 25 μL of 3M NaAc and 1 mL of isopropanol were introduced into the solution, which was then placed at -80 °C to allow for precipitation for 30 minutes. The precipitate was resuspended in water and subjected to purification via high performance liquid chromatography. All synthesized RNA molecules were characterized through MALDI-TOF (Shimadazu Biotech Axima Performance). The ligation of pseudo-uridine modified short RNA precursors and the purification of pseudo-uridien modified circRNA were the same as mentioned above. The sequences of pseudo-uridine modified circRNA were shown in Table S6.

Supporting Figures

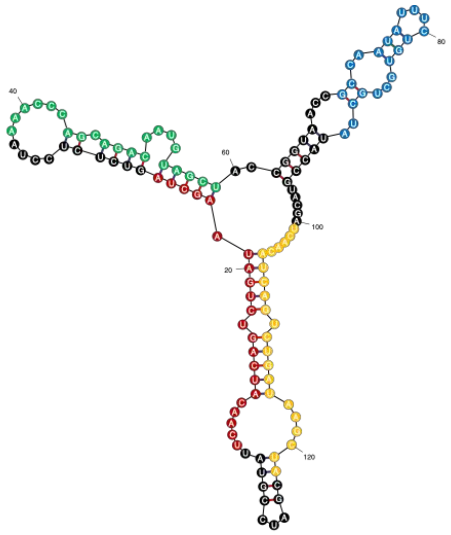

Figure S1 Predicted secondary structure of circSPONGE.

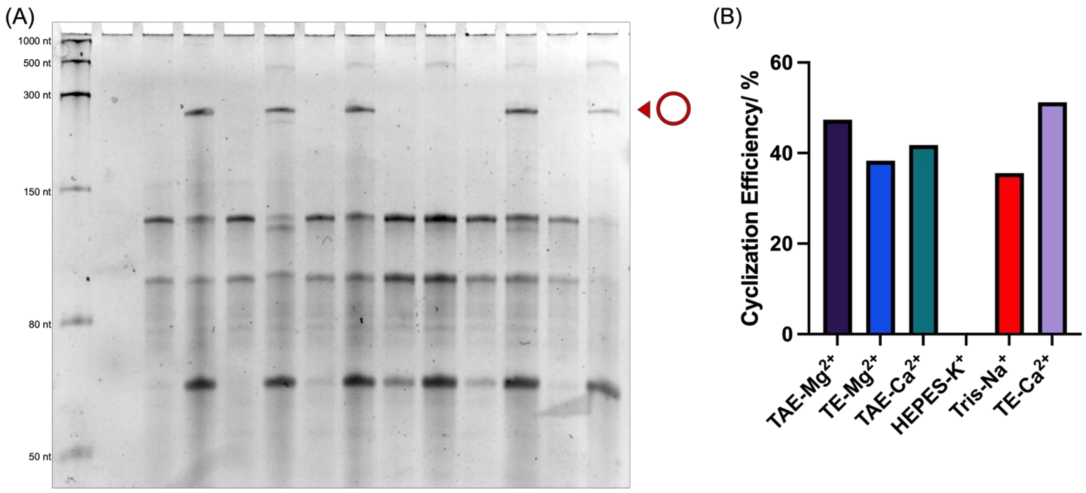

Figure S2 Optimization of assembly buffer. (A) 10% denaturing PAGE analysis of linear ligation and circular ligation in different assembly buffer. Lane 1 to 14: low range ssRNA ladder; no enzyme control; linear ligation in TAE-Mg^{2+}(12.5 mM); circular ligation in TAE-Mg^{2+}(12.5 mM); linear ligation in TE-Mg^{2+}(5 mM); circular ligation in TE-Mg^{2+}(5 mM); linear ligation in TAE-Ca^{2+}(10 mM); circular ligation in TAE-Ca^{2+}(10 mM); linear ligation in HEPES-K^{+}(300 mM); circular ligation in HEPES-K^{+}(300 mM); linear ligation in Tris-Na^{+}(50 mM); circular ligation in Tris-Na^{+}(50 mM); linear ligation in TE-Ca^{2+}(10 mM); circular ligation in TE-Ca^{2+}(10 mM). (B) Quantitative analysis of cyclization efficiency through Image J.

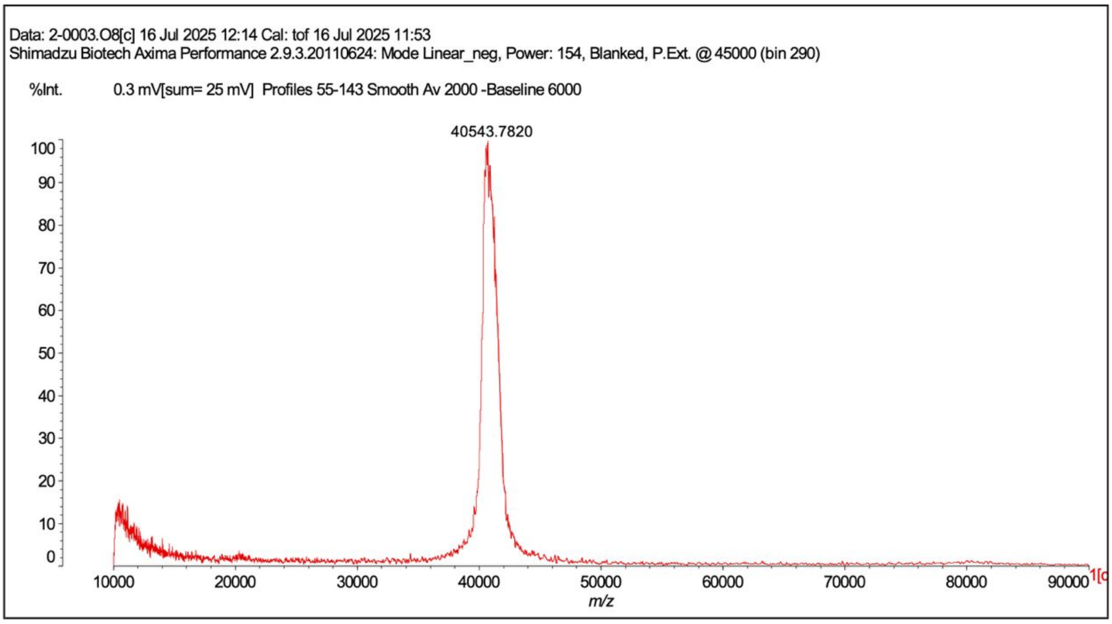

Figure S3 MALDI-TOF result of circSPONGE.

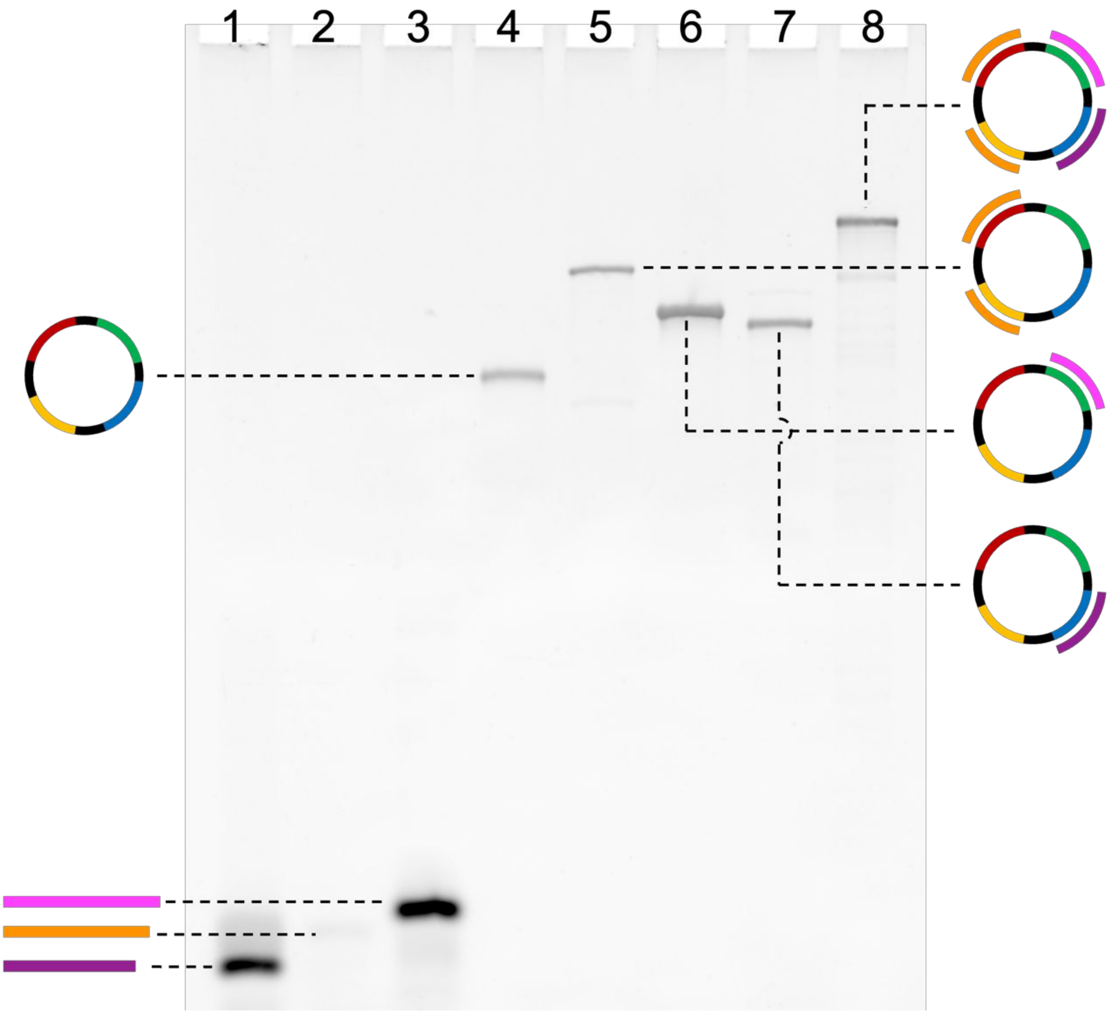

Figure S4 8% native PAGE analysis of the binding of miRNAs to circSPONGE. Lane 1 to 8: miR-195; miR-21; miR-221; circSPONGE; circSPONGE and miR-21; circSPONGE and miR-221; circSPONGE and miR-195; circSPONGE and miR-21, miR-221 and miR-195.

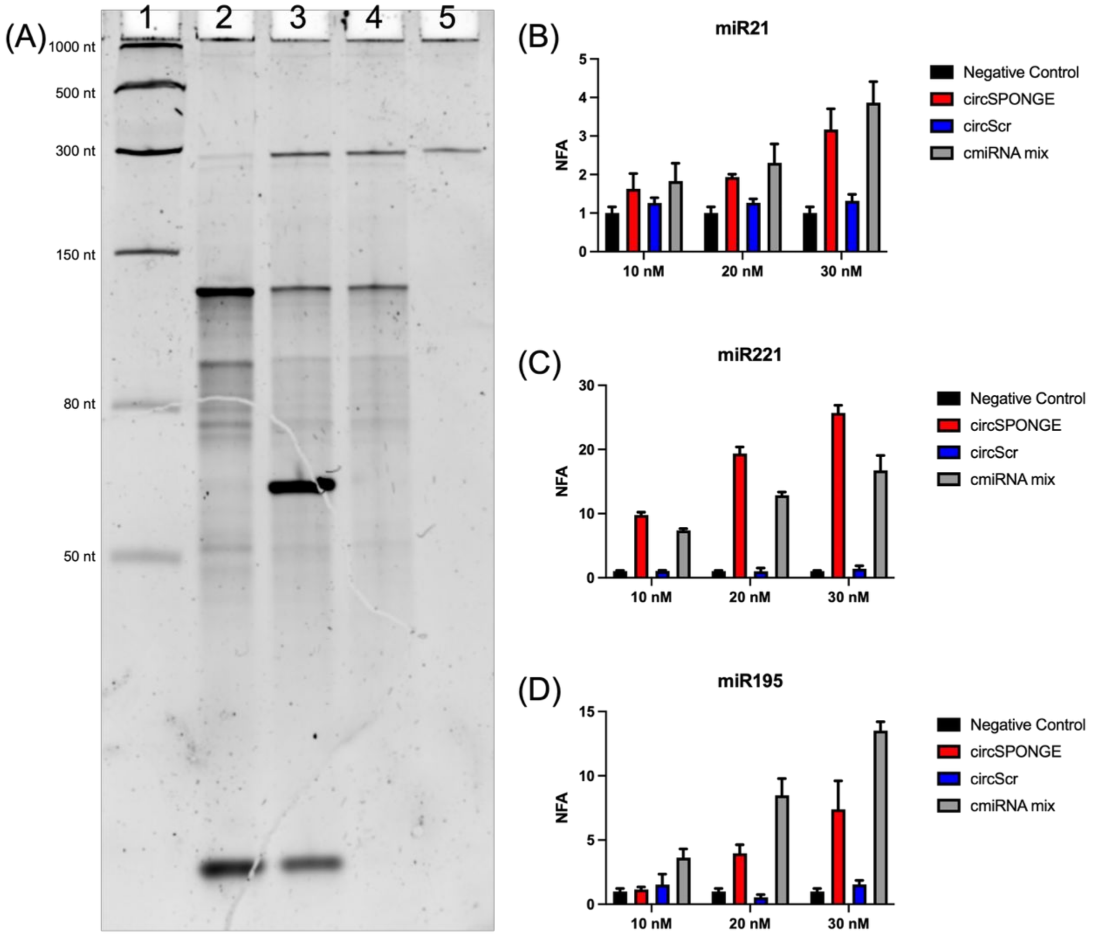

Figure S5 Synthesis and dual-luciferase reporter results of a circSPONGE variant with two binding sites for miR-221, and single binding sites for miR-21 and one for miR-195. (A) The characterization of the synthesis and purification of circSPONGE through 10% denaturing PAGE. Lane 1-5: low range ssRNA ladder; linear ligation; circular ligation product; DNase I digestion product; and RNase R digestion product. (B)-(D) Dual-luciferase reporter results for miR-21 (B), miR-221 (C), and miR-195 (D) in MCF-7 cells.

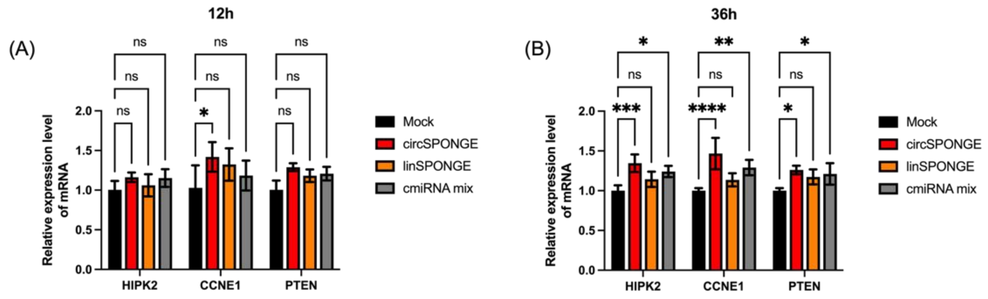

Figure S6 Relative expression levels of downstream mRNAs at 12 h (A) and 48 h (B) after transfection with 30 nM miRNA inhibitors (circSPONGE, linSPONGE and cmiRNA mix). The mRNA expression levels were measured through RT-qPCR using GAPDH mRNA as internal control, at least three independent experiments were performed, and multiple comparisons were performed using one-way ANOVA with Tukey's test. *P < 0.05, **P < 0.01, and ***P < 0.001, ****P < 0.0001.

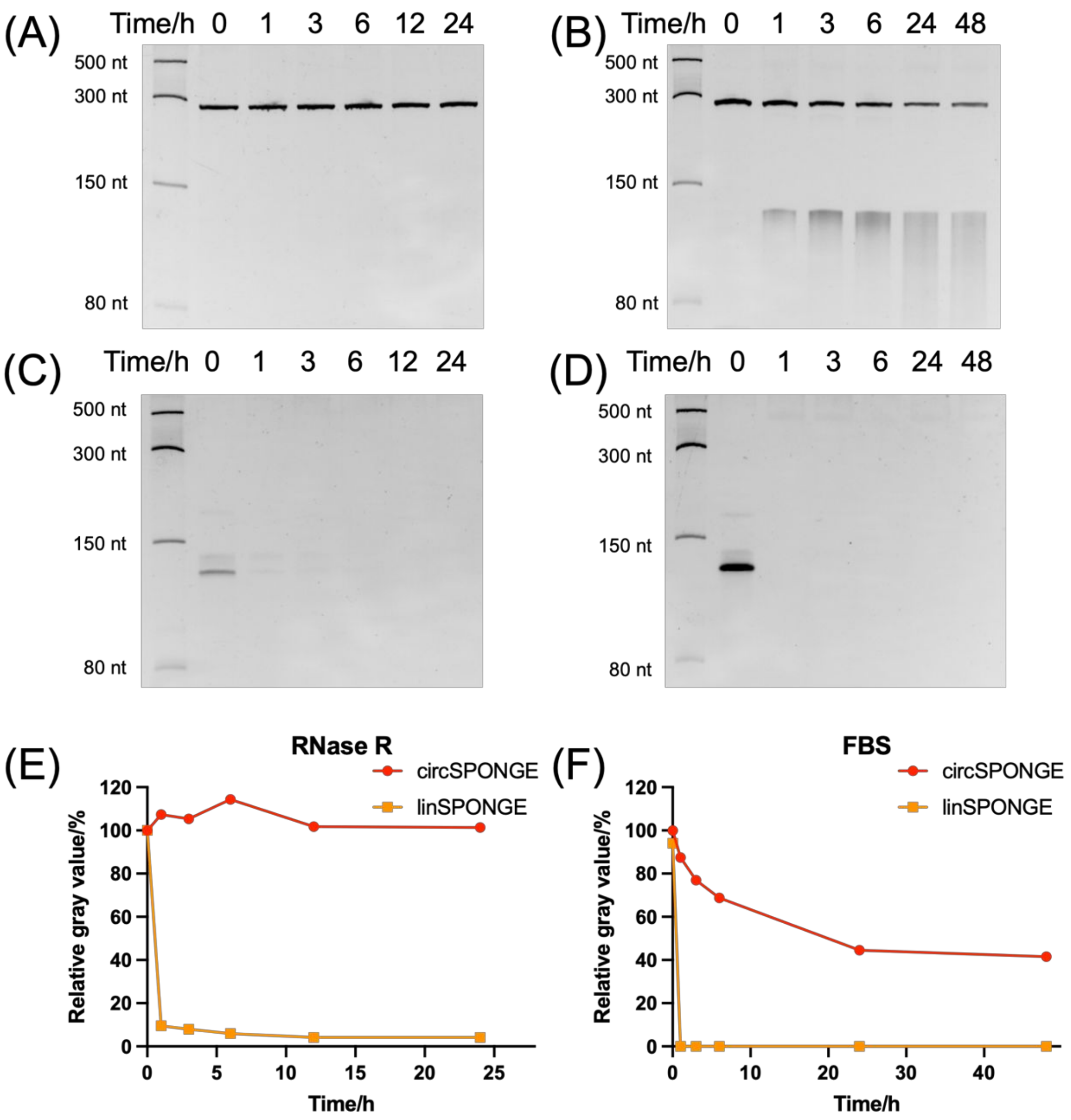

Figure S7 Biostability test of circSPONGE and linSPONGE. (A, B) 10% denaturing PAGE analysis of circSPONGE after incubating with RNase R (A) or 10% FBS (B) at 37 °C. (C, D)10% denaturing PAGE analysis of linSPONGE after incubating with RNase R (C) or 10% FBS (D) at 37 °C. (E) Quantitative analysis of the stability of circSPONGE and linSPONGE in RNase R. (F) Quantitative analysis of the stability of circSPONGE and linSPONGE in 10% FBS.

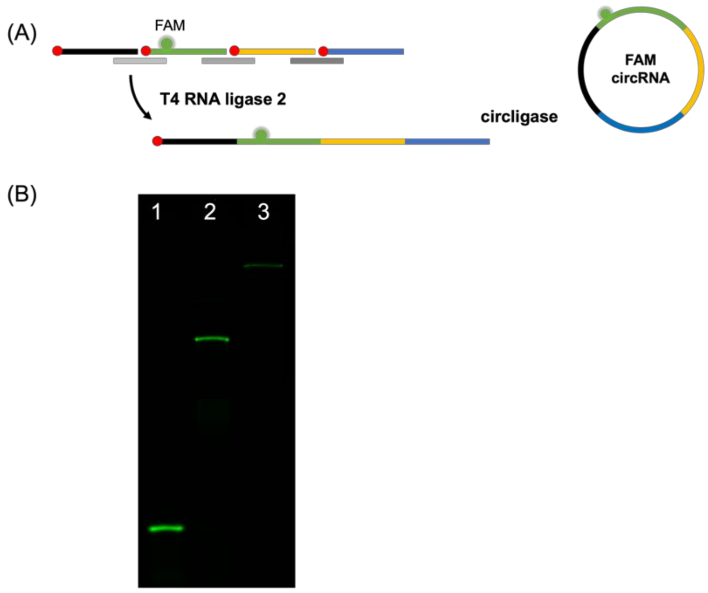

Figure S8 Synthesis of FAM-labeled circSPONGE. (A) Synthesis route of FAM-labeled circSPONGE. (B) 10% denaturing PAGE analysis of the synthesis and purification of FAM-labeled circSPONGE. Lane 1 to 3: FAM-cirmir-221; linear ligation; circular ligation.

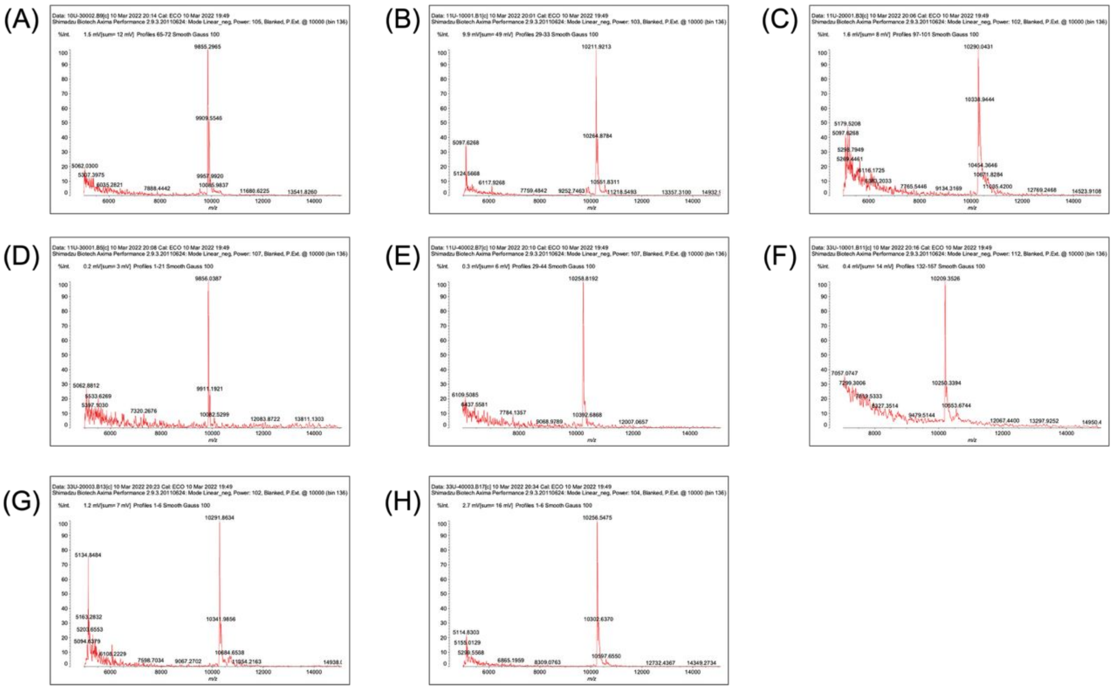

Figure S9 MALDI-TOF result of synthesized pseudo-uridine modified short RNA precursors. (A)10Ψ-3. (B)11Ψ-1. (C)11Ψ-2. (D)11Ψ-3. (E)11Ψ-4. (F)33Ψ-1. (G)33Ψ-2. (H)33Ψ-4.

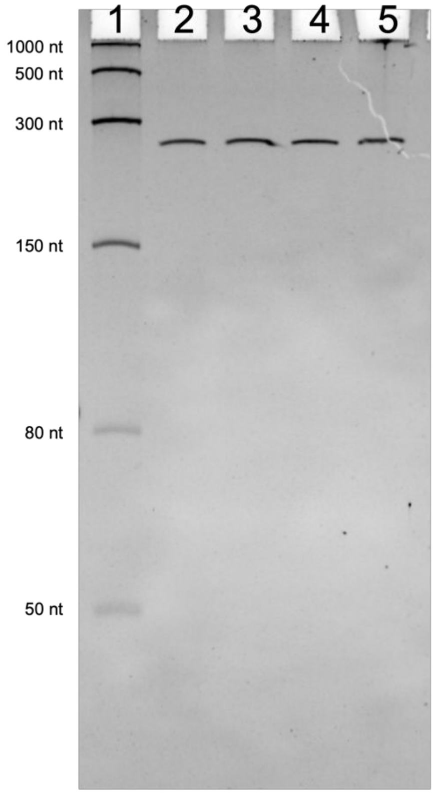

Figure S10 10% denaturing PAGE analysis of pseudo-uridine modified circSPONGE. Lane 1 to 5: low range ssRNA ladder; circSPONGE; circ-10Ψ; circ-11Ψ; circ-33Ψ.

Supporting Tables

Table S1 Sequence for the synthesis of circSPONGE.

Name	Sequence	Length	Modification
cirmir-21	CGUAUUCAACAUCAGUCUGAUAAGCUAGUCUC	32	5'-P
cirmir-221	UCCUAAAACCCAGCAGACAAUGUAGCUACCGG	32	5'-P
cirmir-195	UAACCGCCAAUAUUUCUGUGCUGCUAUACCG	31	5'-P
cirmir-21B	UACGAUCAACAUCAGUCUGAUAAGCUACGAUC	32	5'-P
DNA-C1	GGTTTTAGGAGAGACTAGCT	20	-----
DNA-C2	TTGGCGGTTACCGGTAGCTA	20	-----
DNA-C3	GTTGATCGTACGGTATAGCA	20	-----
cycle-A	GATGTTGATCGTACGGGGAGAGACTAGCTTATCAGACTGATGTTGAATCGTAGCTTATCAGACT	64	-----
circSPONGE	CGUAUUCAACAUCAGUCUGAUAAGCUAGUCUCUCCUAAAACCCAGCAGACAAUGUAGCUACCGGUAACCGCCAAUAUUUCUGUGCUGCUAUACCGUACGAUCAACAUCAGUCUGAUAAGCUACGAUC	127	Circular

Tabel S2 Sequence for the synthesis of circScr.

Name	Sequence	Length	Modification
scr-R-1	CGUAUCAUUAAUGUCGGACAACUCAA UGUCUC	32	5'-P
scr-R-2	UCCUAUUCUCCGAACGUGUCACGUUU ACCGG	32	5'-P
scr-R-3	UAACCACAGGUUGAUAGCGCCAAUAA UACCG	31	5'-P
scr-R-4	UACGACAUUAAUGUCGGACAACUCAA UCGAUC	32	5'-P
Scr-C1	GAGAATAGGAGAGACATTGA	20	-----
Scr-C2	CCTGTGGTTACCGGTAAACG	20	-----
Scr-C3	TAATGTCGTACGGTATTATT	20	-----
Cycle-Scr-R	CATTAATGTCGTACGGGGAGAGACATT GAGTTGTCCGACATTAATGATCGATTG AGTTGTCCGA	64	-----
circScr	CGUAUCAUUAAUGUCGGACAACUCAA UGUCUCUCCUAUUCUCCGAACGUGUC ACGUUUACCGGUAACCACAGGUUGAU AGCGCCAAUAAUACCGUACGACAUUA AUGUCGGACAACUCAAUCGAUC	126	Circular

Table S3 Sequences for the short RNA complementary to miRNA

Name	Sequence	Length
miR-21	UAGCUUAUCAGACUGAUGUUGA	22
miR-221	AGCUACAUUGUCUGCUGGGUUUC	23
miR-195	UAGCAGCACAGAAAUAUUGGC	21
cmiR-21	UCAACAUCAGUCUGAUAAGCUA	22
cmiR-221	UAAACCCAGCAGACAAUGUAGCU	23
cmiR-195	GCCAAUAUUUCUGUGCUGCUA	21

Table S4 Primers for RT-qPCR

Name	Sequence
hPTEN-Fwd	TGAGTTCCCTCAGCCGTTACCT
hPTEN-Rev	GAGGTTTCCTCTGGTCCTGGTA
hHIPK2-Fwd	AGCGTCATCACCATCAGCAGTG
hHIPK2-Rev	AGTCGTGGACTGTGACACAGCT
hCCNE1-Fwd	GTGTGGGAGCCAGCCTTG
hCCNE1-Rev	ATCATCTTCTTTGTCAGGTGTGG
hGAPDH-Fwd	GGAGCGAGATCCCTCCAAAAT
hGAPDH-Rev	GGCTGTTGTCATACTTCTCATGG
mGAPDH-Fwd	AGGTCGGTGTGAACGGATTTG
mGAPDH-Rev	GGGGTCGTTGATGGCAACA
mIL-6-Fwd	CTGCAAGAGACTTCCATCCAG
mIL-6-Rev	AGTGGTATAGACAGGTCTGTTGG
mTNF-α-Fwd	CAGGCGGTGCCTATGTCTC
mTNF-α-Rev	CGATCACCCCGAAGTTCAGTAG
mIL-1β-Fwd	GAAATGCCACCTTTTGACAGTG
mIL-1β-Rev	TGGATGCTCTCATCAGGACAG
mIFN-β-Fwd	CAGCTCCAAGAAAGGACGAAC
mIFN-β-Rev	GGCAGTGTAACTCTTCTGCAT
mCCL2-Fwd	TAGGCTGGAGAGCTACAAGAGG
mCCL2-Rev	AGTGCTTGAGGTGGTTGTGG

Tabel S5 Sequences for the synthesis of fluorescent modified circRNA

Name	Sequence	Length	Modification
FAM-cirmir-221	UCC/iFAMdT/AAAACCCAGCAGACAAUGUAGCUACCGG	32	5'-P, iFAMdT
Cy3-cirmir-195	UAACCGCCAAUAU/iCy3dT/UCUGUGCUGCUAUACCG	31	5'-P, iCy3dT
Cy5-cirmir-21B	UACGA/iCy5dT/CAACAUCAGUCUGAUAAGCUACGAUC	32	5'-P, iCy5dT

Table S6 Sequences for the synthesis of pseudo-uridine modified circRNA

Name	Sequence	Length	Modification
11Ψ-1	CGΨAUUCAACAUCAGΨCΨGAUAAGC UAGΨCUC	32	5'-P, pseudoU
11Ψ-2	UCCUAAAACCCAGCAGACAAΨGΨAG CUACCGG	32	5'-P, pseudoU
11Ψ-3	UAACCGCCAAUAUUUCΨGΨGCΨGCU AUACCG	31	5'-P, pseudoU
11Ψ-4	UACGAUCAACAUCAGΨCΨGAUAAGC UACGAUC	32	5'-P, pseudoU
10Ψ-3	ΨAACCGCCAAΨAΨΨΨCΨGΨGCΨGC ΨAΨACCG	31	5'-P, pseudoU
33Ψ-1	CGΨAΨΨCAACAΨCAGΨCΨGAΨAAG CΨAGΨCΨC	32	5'-P, pseudoU
33Ψ-2	ΨCCΨAAAACCCAGCAGACAAΨGΨAG CΨACCGG	32	5'-P, pseudoU
33Ψ-4	ΨACGAΨCAACAΨCAGΨCΨGAΨAAG CΨACGAΨC	32	5'-P, pseudoU
circ-10Ψ	CGUAUUCAACAUCAGUCUGAUAAGC UAGUCUCUCCUAAAACCCAGCAGAC AAUGUAGCUACCGGΨAACCGCCAAΨ AΨΨΨCΨGΨGCΨGCΨAΨACCGUACG AUCAACAUCAGUCUGAUAAGCUACG AUC	127	circular, pseudoU
circ-11Ψ	CGΨAUUCAACAUCAGΨCΨGAUAAGC UAGΨCUCUCCUAAAACCCAGCAGAC AAΨGΨAGCUACCGGUAACCGCCAAU AUUUCΨGΨGCΨGCUAUACCGUACGA UCAACAUCAGΨCΨGAUAAGCUACGA UC	127	circular, pseudoU
circ33Ψ	CGΨAΨΨCAACAΨCAGΨCΨGAΨAAG CΨAGΨCΨCΨCCΨAAAACCCAGCAGA CAAΨGΨAGCΨACCGGΨAACCGCCAA ΨAΨΨΨCΨGΨGCΨGCΨAΨACCGΨAC GAΨCAACAΨCAGΨCΨGAΨAAGCΨA CGAΨC	127	circular, pseudoU

Table S7 Sequence of circSPONGE with two miRNA binding sites for miR-221, one for miR-21 and one for miR-195

Name	Sequence	Length	Modification
cirmir-21	CGUAUUCAACAUCAGUCUGAUAAGCU AGUCUC	32	5'-P
cirmir-221	UCCUAAAACCCAGCAGACAAUGUAGC UACCGG	32	5'-P
cirmir-195	UAACCGCCAAUAUUUCUGUGCUGCUA UACCG	31	5'-P
cirmir-221B	UACGAAAACCCAGCAGACAAUGUAGC UCGAUC	32	5'-P
DNA-C1	GGTTTTAGGAGAGACTAGCT	20	-----
DNA-C2	TTGGCGGTTACCGGTAGCTA	20	-----
DNA-C3	GGTTTTCGTACGGTATAGCA	20	-----
cycle-A	CTGGGTTTTCGTACGGGGAGAGACTAG CTTATCAGACTGATGTTGAATCGAGCT ACATTGTCTG	64	-----
circSPONGE-New	CGUAUUCAACAUCAGUCUGAUAAGCU AGUCUCUCCUAAAACCCAGCAGACAA UGUAGCUACCGGUAACCGCCAAUAUU UCUGUGCUGCUAUACCGUACGAAAAC CCAGCAGACAAUGUAGCUCGAUC	127	Circular
